# Post-styloid parapharyngeal neurogenic tumors: imaging-based prediction of nerve origin and clinical outcomes — a single-center retrospective case series

**DOI:** 10.3389/fsurg.2026.1775746

**Published:** 2026-06-23

**Authors:** Pei-Han Liu, Hsiu-San Hsu, Chun-Hung Hua, Li-An Su, Wan-Ling Yi, Yung-An Tsou, Chia-Der Lin, Liang-Chun Shih, Chien-Chi Lu

**Affiliations:** 1 Graduate Institute of Biomedical Sciences, China Medical University, Taichung, Taiwan; 2 Department of Otorhinolaryngology-Head and Neck Surgery, China Medical University Hospital, Taichung, Taiwan; 3 Department of Otorhinolaryngology-Head and Neck Surgery, Asia University Hospital, Taichung, Taiwan; 4 Department of Nursing, Hungkuang University, Taichung, Taiwan; 5 School of Medicine, China Medical University, Taichung, Taiwan

**Keywords:** nerve of origin prediction, neurogenic tumors, post-styloid parapharyngeal space, stereotactic radiosurgery, vessel displacement patterns

## Abstract

**Introduction:**

Post-styloid parapharyngeal space (PPS) tumors are rare and predominantly neurogenic. Because tumors arising from the vagus nerve or the cervical sympathetic chain are associated with distinct postoperative morbidities, accurate preoperative identification of the nerve of origin is clinically important for surgical planning and risk counseling. The primary endpoint was the accuracy of imaging-based nerve-of-origin prediction using vessel displacement patterns, compared with intraoperative confirmation.

**Methods:**

We retrospectively reviewed adult patients with post-styloid PPS tumors treated at a single center between 1998 and 2023. Vessel displacement patterns on computed tomography (CT) or magnetic resonance imaging (MRI) were used to estimate nerve of origin and were verified intraoperatively in surgically treated patients. Management strategy, pathology (when available), complications, and follow-up outcomes were recorded.

**Results:**

Fifteen patients (mean age 44.5 years; 67% male) were identified. A painless neck mass was the most common presenting symptom (53%). Imaging suggested a vagal origin in 11 tumors and a sympathetic chain origin in 4 tumors. Among the 9 surgically treated patients with intraoperative confirmation, imaging correctly predicted the nerve of origin in 8 cases (88.9%). One tumor predicted as vagal in origin was intraoperatively identified as arising from the sympathetic chain and was complicated by postoperative Horner's syndrome. Surgical resection was performed in 9 patients, stereotactic radiosurgery in 2, and active surveillance in 4. Postoperative vocal fold palsy and Horner's syndrome each occurred in one patient. No recurrence or progression was documented during available follow-up.

**Discussion:**

Routine cross-sectional imaging of vessel displacement patterns may provide a valuable preoperative estimate of nerve origin for post-styloid PPS neurogenic tumors. This approach may help surgeons individualize treatment strategies and risk assessment. Future multicenter studies with larger surgically verified cohorts are needed to confirm these results.

## Introduction

Parapharyngeal space (PPS) tumors are rare, accounting for 0.5%–1% of all head and neck neoplasms (1). The PPS extends from the skull base to the hyoid bone and is divided into pre- and post-styloid compartments. The post-styloid compartment is clinically significant due to its dense neurovascular structures, including the internal carotid artery (ICA), internal jugular vein (IJV), and cranial nerves IX–XII (2, 3). Neurogenic tumors—primarily schwannomas and paragangliomas—are the most common lesions in this space (4). This study specifically focuses on neurogenic lesions.

Patients with post-styloid PPS tumors typically present with a painless neck mass, although some may develop neurological symptoms such as cranial nerve palsy, dysphagia, or otalgia. Early diagnosis is challenging owing to the deep anatomical location of these tumors, and determining the nerve of origin preoperatively is often difficult. Accurate identification of the tumor's nerve of origin is critical for selecting the optimal surgical approach and minimizing neurovascular complications. Vagal schwannomas typically arise from the vagus nerve and may result in postoperative vocal fold paralysis, whereas sympathetic chain schwannomas can lead to Horner's syndrome—characterized by ptosis, miosis, and anhidrosis—when the sympathetic trunk is involved (5). Accordingly, nerve-specific preoperative assessment aids in anticipating functional outcomes and in planning nerve-preserving surgical strategies.

Computed tomography (CT) and magnetic resonance imaging (MRI) are widely used for localization, and characteristic vessel-displacement patterns have been proposed to suggest the likely nerve of origin (6). Cervical sympathetic schwannomas typically displace the ICA and IJV laterally, whereas vagal schwannomas separate the ICA or common carotid artery from the IJV, usually shifting the ICA anteromedially. Furukawa et al. proposed that vagal schwannomas tend to splay the ICA away from the IJV, unlike sympathetic schwannomas (7). These imaging patterns provide important preoperative clues to the likely nerve of origin (8).

Surgical resection is generally considered the standard treatment for post-styloid PPS tumors (5); however, the close relationship between these tumors and adjacent neurovascular structures contributes to a substantial risk of postoperative nerve deficits. This reinforces the clinical importance of reliable preoperative assessment—not only for localizing the tumor but also for guiding operative strategies aimed at preserving nerve function. Nonetheless, because these tumors are rare and associated with considerable morbidity, comprehensive institutional experiences with long-term imaging–surgical correlation and treatment decision-making remain limited.

We retrospectively reviewed post-styloid PPS neurogenic tumors treated over a 25-year period. We hypothesized that established ICA–IJV displacement patterns on routine CT/MRI can provide a clinically useful preoperative estimate of the nerve of origin. Accordingly, we aimed to (1) quantify prediction accuracy against intraoperative confirmation in surgically treated patients and (2) describe real-world management pathways (surgery, stereotactic radiosurgery, and observation) and associated outcomes.

## Materials and methods

This retrospective case series was approved by the Institutional Review Board of China Medical University Hospital (approval number: CMUH114-REC2-168). We analyzed patients diagnosed with post-styloid PPS tumors at China Medical University Hospital, Taichung, Taiwan, between January 1998 and December 2023. Eligible cases were identified through electronic medical records, and inclusion required: age ≥ 18 years, radiologically confirmed post-styloid PPS tumors on head and neck computed tomography (CT) or magnetic resonance imaging (MRI), and complete clinical and imaging data. There were no restrictions regarding tumor size, presenting symptoms, or preoperative neurological status; all consecutive adult patients who met these criteria during the study period were included. Patients with pre-styloid tumors, incomplete or non-definitive imaging, or lesions confirmed preoperatively or intraoperatively to be non-neurogenic (e.g., metastatic or inflammatory) were excluded.

Demographic characteristics, clinical presentation, radiologic findings, surgical approaches, and histopathological diagnoses were obtained from electronic medical records, radiology reports, surgical notes, and pathology reports. All preoperative imaging was reviewed independently by two board-certified neuroradiologists with >10 years of experience. Interpretations were made without access to operative or histopathologic data to minimize bias. A standardized imaging review protocol was applied. Preoperative CT/MRI were evaluated for tumor compartment, vessel displacement patterns, skull base extension, enhancement patterns, and vascularity. Interobserver agreement between the two radiologists for the imaging-based prediction of nerve origin was assessed using Cohen's kappa coefficient. Disagreements were resolved by consensus review. When feasible, fine-needle aspiration (FNA) was performed to exclude malignancy; however, its use was limited by deep anatomical location and neurovascular risk. Nerve of origin was predicted primarily based on established displacement criteria. For non-surgically managed cases, lesions were classified as presumed neurogenic based on their post-styloid compartment location, vessel displacement patterns, typical CT/MRI features, and clinical-radiologic follow-up, while acknowledging that histopathologic confirmation was unavailable and that selected differential diagnoses, including minor or ectopic salivary gland tumors, could not be completely excluded in some cases.

Treatment decisions were made at multidisciplinary case conferences based on tumor operability, skull base involvement, symptomatology, and patient comorbidity and preference. For surgically treated patients, the operative approach was tailored to tumor size, skull base extension, proximity to major vessels and the mandible, and the anticipated nerve of origin. Intraoperative neurophysiological monitoring was used in all surgically treated cases. Depending on tumor location, surgical exposure, and equipment availability, monitoring consisted of electromyographic monitoring, direct nerve stimulation, or both. For suspected vagal schwannomas, intracapsular enucleation via internal decompression and meticulous capsular dissection was attempted whenever a definable plane existed. Conversely, for tumors originating from the sympathetic chain, *en bloc* resection was performed if the mass was inseparable from the trunk or lacked a functional cleavage plane. Ultimately, the extent of resection was strictly guided by intraoperative findings to maximize functional preservation. Surgery was offered to operable patients with potential for nerve-preserving resection, stereotactic radiosurgery (SRS) to high-risk or skull base–extending tumors, and radiographic surveillance to asymptomatic, radiographically stable lesions, defined as no interval growth on serial imaging.

Post-treatment follow-up was generally performed through clinic visits, typically at 3-month intervals during the first year and semi-annually thereafter when clinically indicated. Follow-up information was obtained through in-person clinic evaluations supplemented by electronic medical record review; no telephone follow-up was used. For patients managed with SRS or observation, follow-up imaging was typically performed at 6–12-month intervals. Imaging studies were repeated when recurrence was clinically suspected or when new neurological symptoms developed. Recurrence was defined by radiologic or histopathologic evidence during follow-up. Patients lost to follow-up were censored at their last documented evaluation.

As this was a retrospective study, the requirement for informed consent for study participation was waived by the Institutional Review Board of China Medical University Hospital (CMUH114-REC2-168). All data were de-identified prior to analysis and reporting in accordance with institutional and ethical guidelines. To further protect patient privacy, potentially identifiable data such as exact ages have been categorized into ranges, and specific pathological diagnoses have been generalized in the summary tables. Statistical analysis was primarily descriptive, with categorical variables presented as frequencies and percentages. Diagnostic performance metrics, including accuracy, sensitivity, specificity, positive predictive value, and negative predictive value, were calculated among surgically confirmed cases only. Interobserver agreement was assessed using Cohen's kappa coefficient. Sample size was determined by the number of eligible patients identified during the study period. Given the retrospective, single-center design and limited sample size, potential selection and reporting bias could not be fully excluded.

## Results

During the study period, 15 patients with post-styloid PPS tumors were identified at our medical center, including both outpatient and inpatient cases. Table 1 presents the demographic and clinical characteristics. The mean (± SD) age was 44.53 ± 15.07 years (range, 20–65), and 10 patients were male. A painless neck mass was the most common presenting symptom (8/15, 53%), followed by local numbness (3/15, 20%).

**Table 1:** Clinical characteristics of patients with post-styloid parapharyngeal space tumors.

Characteristic	Patients (*N* = 15)
Mean age (range) — year	44.53 ± 15.07 (20–65)
Sex — no. (%)	
Male	10 (67)
Female	5 (33)
Symptoms — no. (%)	
Neck mass	10 (67)
Painful	2 (13)
Painless	8 (53)
Local numbness	3 (20)
Lump sensation of the throat	2 (13)
Voice change	2 (13)
Trismus	1 (7)
Sore throat	1 (7)
Headache	1 (7)
Frequent coughing	1 (7)
Location — no. (%)	
Right neck	9 (60)
Left neck	5 (33)
Bilateral	1 (7)
Fine needle aspiration — no. (%)	
Suspected malignancy (atypia)	1 (7)
Benign	5 (33)
Management — no. (%)	
Surgical resection	9 (60)
Transmandibular approach	3/9 (33)
Transcervical approach	6/9 (67)
Stereotactic radiosurgery	2 (13)
Observation	4 (27)
Postoperative complications among surgically treated patients — no./total (%)	
Vocal fold palsy	1/9 (11)
Horner's syndrome	1/9 (11)
Recurrence — no. (%)	0 (0)
Follow-up duration— months (mean ± SD; range)	30.5 ± 39.3 (1–132)

Data are presented as number of patients and percentage of the total cohort (*N* = 15), unless otherwise indicated. Surgical approaches and postoperative complications are reported among surgically treated patients (*n* = 9). Recurrence is reported among treated patients, including those who underwent surgery or stereotactic radiosurgery (*n* = 11). Symptoms were not mutually exclusive.

**Figure 1:**
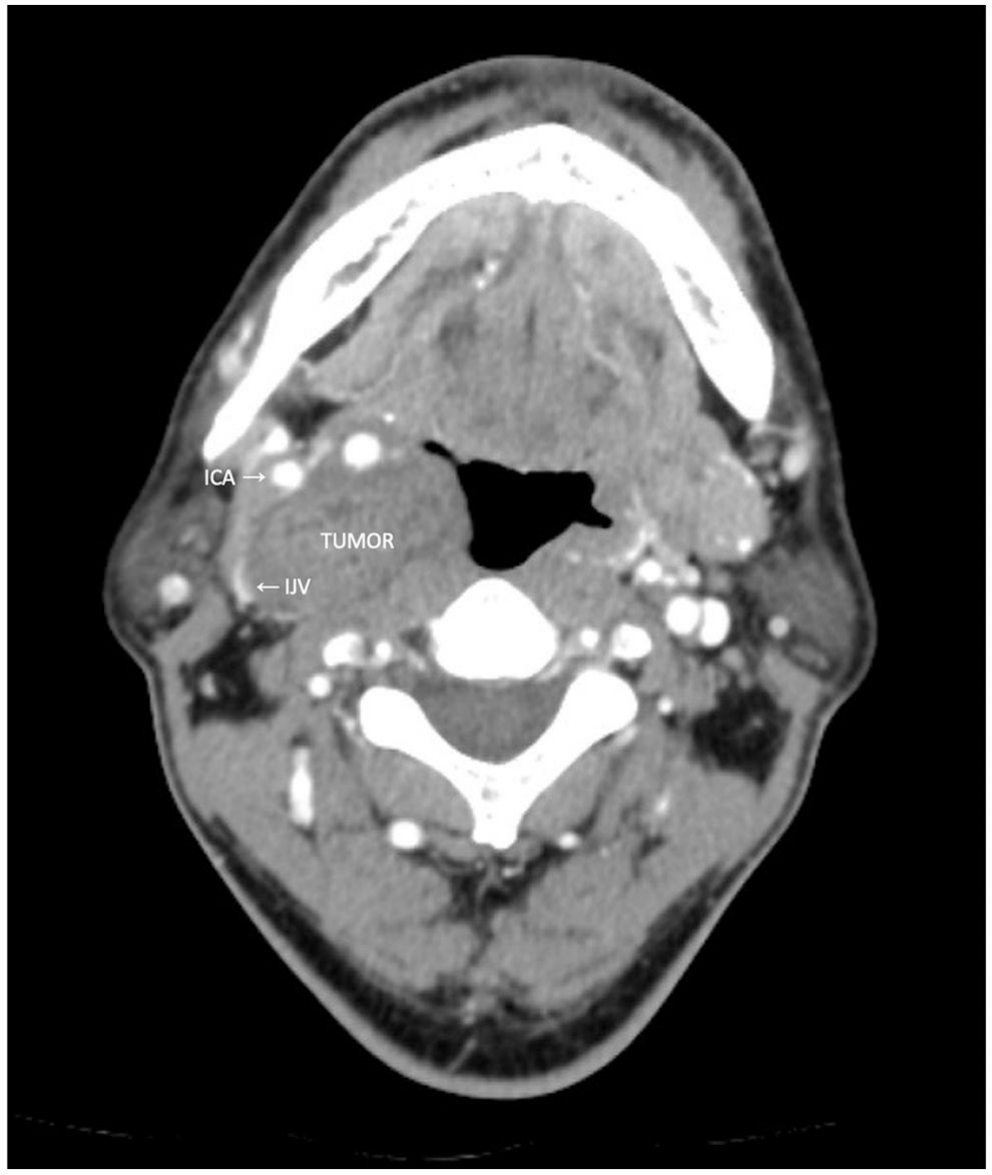
Imaging findings of a vagal schwannoma. Axial contrast-enhanced CT image of the neck shows a well-circumscribed post-styloid parapharyngeal space mass causing separation of the internal carotid artery (ICA) and internal jugular vein (IJV), with anteromedial displacement of the ICA. This vessel displacement pattern was interpreted as suggestive of vagal nerve origin. Intraoperative findings confirmed tumor adherence to the vagus nerve.

**Figure 2:**
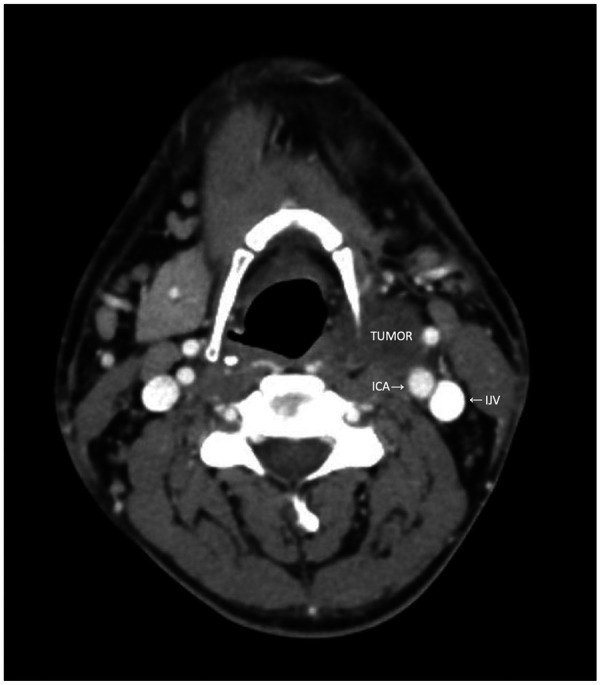
Imaging findings of a sympathetic chain schwannoma. Axial contrast-enhanced CT image of the neck shows a post-styloid parapharyngeal space mass displacing the internal carotid artery (ICA) and internal jugular vein (IJV) together laterally without clear separation between the two vessels. This pattern was interpreted as suggestive of sympathetic chain origin, which was confirmed intraoperatively.

All patients underwent preoperative head and neck imaging, including CT (*n* = 12) or MRI (*n* = 3). Most tumors demonstrated well-demarcated borders and mild contrast enhancement. Eleven tumors showed splaying of the ICA and IJV, a pattern commonly associated with vagal schwannomas (Figure 1). Of these, 10 demonstrated anteromedial ICA displacement. Four tumors exhibited displacement patterns consistent with sympathetic chain schwannomas, characterized by lateral displacement of both the ICA and IJV without splaying (Figure 2). Table 2 summarizes the correlation between imaging-based predictions and intraoperative findings. Interobserver agreement between the two radiologists for imaging-based nerve-of-origin prediction was perfect, with a Cohen's kappa coefficient of 1.00.

**Table 2:** Case-by-case comparison of imaging-based predicted tumor origin, management strategy, pathology, and intraoperative confirmation.

	Age (year-old)	Sex	Preoperative suspected tumor origin	Management	Pathological diagnosis	Intraoperative findings
1	20–29	M	Vagus nerve	Transmandibular	Schwannoma	Vagus nerve
2	20–29	M	Vagus nerve	Observation	N/A	N/A
3	40–49	F	Vagus nerve	Transcervical	Paraganglioma	Vagus nerve
4	30–39	F	Vagus nerve	Transmandibular	Schwannoma	Vagus nerve
5	30–39	M	Vagus nerve	Transcervical	Schwannoma	Vagus nerve
6	60–69	M	Vagus nerve	Stereotactic radiosurgery	N/A	N/A
7	60–69	M	Sympathetic chain	Transcervical	Schwannoma	Sympathetic chain
8	30–39	M	Vagus nerve	Stereotactic radiosurgery	N/A	N/A
9	30–39	M	Sympathetic chain	Transmandibular	Schwannoma	Sympathetic chain
10	50–59	F	Vagus nerve	Transcervical	Schwannoma	Vagus nerve
11	60–69	F	Sympathetic chain	Transcervical	Schwannoma	Sympathetic chain
12	20–29	F	Sympathetic chain	Observation	N/A	N/A
13	60–69	M	Vagus nerve	Observation	N/A	N/A
14	50–59	M	Vagus nerve	Observation	N/A	N/A
15	50–59	M	Vagus nerve	Transcervical	Schwannoma	Sympathetic chain

M, male; F, female; N/A, not applicable (patients who did not undergo surgery; thus, pathological diagnosis and intraoperative findings were unavailable). *N* = 15.

Six of the 15 patients (40%) underwent preoperative FNA, including one case suspicious for malignancy. FNA was not routinely performed because of the deep location and associated neurovascular risk.

Nine patients (60%) underwent surgical treatment. Among these surgically treated patients, transcervical and transmandibular approaches were used in 6 (66.7%) and 3 (33.3%) patients, respectively. Intracapsular enucleation was performed when a nerve-preserving dissection plane was identified, particularly for tumors suspected to arise from the vagus nerve. En bloc resection was performed when the tumor was inseparable from the sympathetic chain or when functional nerve preservation was not feasible. Intraoperative neurophysiological monitoring was used to assist in identifying functional neural structures and preserving nerve function. Depending on the case, monitoring consisted of electromyographic monitoring, direct nerve stimulation, or both.

**Figure 3:**
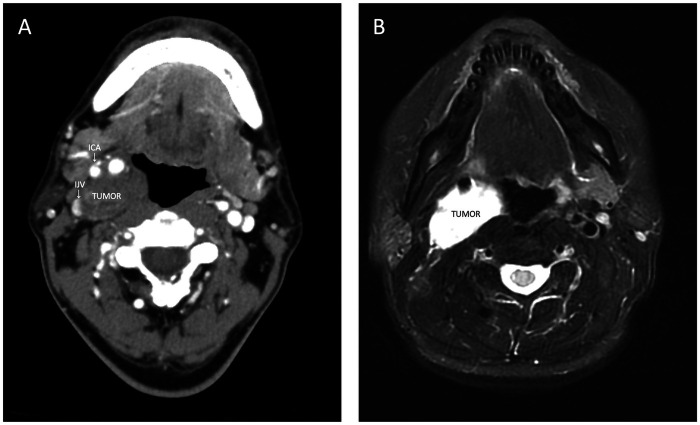
Non-surgically managed post-styloid parapharyngeal space tumor with a presumed neurogenic origin. **(A)** Axial contrast-enhanced CT image shows a well-demarcated enhancing mass in the right post-styloid parapharyngeal space, splaying the internal carotid artery (ICA) and internal jugular vein (IJV), with anteromedial displacement of the ICA. **(B)** Axial fat-saturated T2-weighted MR image shows a well-circumscribed post-styloid mass. Although the tumor location and vessel displacement pattern strongly favored a neurogenic lesion, a minor or ectopic salivary gland tumor could not be definitively excluded and remained a radiologic differential consideration. The patient was managed with active surveillance, and follow-up imaging demonstrated no interval growth over 32 months.

Two patients (13%) underwent stereotactic radiosurgery (SRS) due to skull base extension and neurological symptoms. Four patients (27%) with asymptomatic and radiographically stable tumors were managed with observation. A representative non-surgically managed case with MRI findings is shown in Figure 3.

Histopathology was available for the 9 surgically treated patients: schwannoma was the most common diagnosis (*n* = 8), and one tumor was a paraganglioma (*n* = 1). Preoperative FNA cytology was performed in 6 patients; however, patients managed with stereotactic radiosurgery or observation had no histopathologic confirmation.

**Figure 4:**
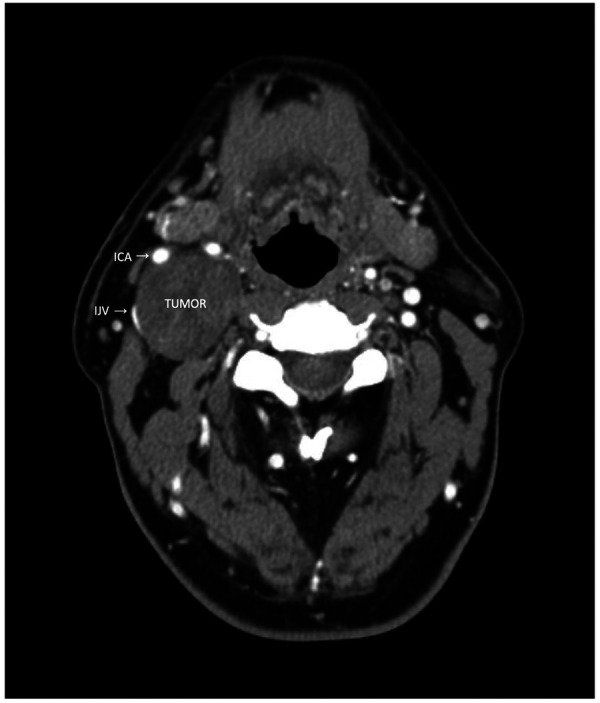
Misclassified case based on vessel displacement pattern. Axial contrast-enhanced CT image of the neck shows separation of the internal carotid artery (ICA) and internal jugular vein (IJV), initially suggesting vagal nerve origin. However, intraoperative findings demonstrated sympathetic chain origin, and the patient developed postoperative Horner's syndrome.

Among the 9 patients who underwent surgical resection, preoperative imaging correctly identified the nerve of origin in eight cases, yielding an overall accuracy of 88.9%. A contingency analysis comparing imaging-based prediction with intraoperative confirmation is shown in Table 3. When vagal origin was considered the positive classification, sensitivity, specificity, positive predictive value, and negative predictive value were 100.0%, 75.0%, 83.3%, and 100.0%, respectively. When sympathetic chain origin was considered the positive classification, the corresponding values were 75.0%, 100.0%, 100.0%, and 83.3%, respectively. One tumor initially interpreted as a vagal schwannoma was intraoperatively identified as arising from the sympathetic chain, resulting in postoperative Horner's syndrome (Figure 4). The six patients who did not undergo surgery (SRS or observation) lacked intraoperative confirmation and were therefore excluded from diagnostic performance calculations. Given the small number of surgically confirmed cases, these metrics were interpreted descriptively.

**Table 3:** Contingency table comparing imaging-based prediction and intraoperative confirmation of nerve origin among surgically treated patients.

Intraoperative confirmed nerve of origin			
Imaging-based predicted nerve of origin	Vagus nerve	Sympathetic chain	Total
Vagus nerve	5	1	6
Sympathetic chain	0	3	3
Total	5	4	9

Only patients with intraoperative confirmation were included in this analysis (*n* = 9). Patients managed non-surgically with stereotactic radiosurgery or observation were excluded because intraoperative confirmation was unavailable (*n* = 6).

Among surgically treated patients, postoperative vocal fold palsy and Horner's syndrome each occurred in 1 of 9 patients (11.1%). During available follow-up, no radiographic or clinically documented recurrence or progression was observed among the 11 treated patients; however, these outcomes are limited by the heterogeneous follow-up duration. Follow-up duration ranged from 1 to 132 months, with a mean of 30.5 months, a median of 12 months, and an interquartile range of 3–43 months.

## Discussion

In this study of 15 patients with post-styloid PPS tumors, the mean age was 44.5 years, with a male predominance of 67%, consistent with previous reports (9). The etiology of this sex difference remains unclear, as no hormonal or environmental factors have been statistically established. A painless neck mass was the most common presenting symptom (53%), also in line with prior studies (1). Neurological manifestations were frequent, reflecting the proximity of these tumors to critical neurovascular structures.

Accurate preoperative prediction of the nerve of origin is clinically important for surgical planning and risk counseling in post-styloid PPS tumors. Using routine cross-sectional CT and/or MRI, vessel displacement patterns—particularly the relationship between the ICA and IJV—may provide useful clues for differentiating vagal from sympathetic chain tumors. In our series, imaging-based prediction achieved an accuracy of 88.9% among surgically confirmed cases. Nevertheless, this estimate should be interpreted cautiously because only nine patients had intraoperative confirmation. One lesion preoperatively misidentified as vagal proved to be of sympathetic chain origin intraoperatively, resulting in postoperative Horner's syndrome. This case underscores that vessel displacement patterns should not be evaluated in isolation, but should be integrated with tumor epicenter, enhancement pattern, vascularity, and skull base extension.

Similar diagnostic challenges have been reported in the literature. Anil et al. described that cervical sympathetic chain schwannomas may occasionally produce vessel displacement patterns resembling those of vagal schwannomas, complicating preoperative diagnosis (10). Graffeo et al. further demonstrated that radiographic criteria, while useful, are not uniformly accurate, with some tumors displaying atypical ICA–IJV relationships that led to misclassification (11). Collectively, these findings indicate that vessel displacement alone may be insufficient in certain cases; large tumor size, vascular distortion, and anatomic variation can obscure typical patterns. Vagal paraganglioma represents another important differential diagnosis that may complicate imaging-based prediction of nerve origin in the post-styloid PPS. Because vagal paragangliomas arise along the vagus nerve within the carotid sheath, they may also produce ICA–IJV separation or anteromedial ICA displacement, thereby mimicking the displacement pattern of vagal schwannoma. However, unlike schwannomas, paragangliomas are typically hypervascular lesions and may demonstrate avid contrast enhancement, internal flow voids, or a “salt-and-pepper” appearance on MRI. Therefore, when a post-styloid PPS tumor shows marked enhancement or prominent vascular features, additional vascular imaging such as contrast-enhanced MRI, MR angiography, or CT angiography may be useful (12).

Building on these findings, future studies incorporating standardized imaging criteria, interobserver validation, and advanced analytic tools may further improve preoperative prediction of nerve origin and facilitate more individualized surgical planning.

From a surgical perspective, the distinction between vagal and sympathetic chain origin is clinically meaningful because the expected functional deficit and nerve-preservation strategy differ between these tumors. Vagal schwannomas may allow intracapsular enucleation or meticulous capsular dissection when a safe plane is present, whereas sympathetic chain schwannomas may be more difficult to separate from the parent trunk because sympathetic fibers are thin and closely associated with the tumor capsule. As a result, sympathetic chain schwannomas have traditionally been managed with *en bloc* resection, and postoperative sympathetic deficits have been reported in more than half of cases (13). Prior studies also suggest nearly universal nerve palsy after radical excision and lower rates with intracapsular dissection, with recurrence rates comparable between the two approaches, supporting the feasibility of function-preserving strategies when a plane is present (5). Intraoperative adjuncts such as neurophysiological monitoring, electromyographic stimulation, direct nerve stimulation, and microscopic dissection may assist in identifying functional fascicles and reducing iatrogenic injury (13). Avoiding sympathetic chain injury remains an important surgical goal because Horner's syndrome may affect patient comfort and postoperative quality of life.

Surgical excision remains the mainstay of treatment, with the choice of approach determined by tumor size, location, superior extension, and relationship to the mandible and skull base. In the present series, only transcervical and transmandibular approaches were used, reflecting the location and extent of the included tumors. Tumors adherent to neurovascular structures may require more extensive resection, increasing the risk of permanent deficits. In our series, one case each of vocal fold palsy and Horner's syndrome occurred, reflecting the inherent risks of nerve involvement.

Less invasive modalities are increasingly considered for selected cases. SRS was selected for two patients with skull base–extending tumors, suggesting its potential role in carefully selected patients who are poor surgical candidates or have anatomically challenging lesions. SRS is generally appropriate for skull base tumors ≤ 3 cm, offering outcomes comparable to surgery with reduced morbidity, although it carries a higher risk of cranial nerve injury than fractionated radiotherapy (RT) and is less effective for mobile tumors below the skull base. Fractionated RT remains suitable for schwannomas of any size (1, 14). For smaller tumors, observation, RT, and surgery may yield comparable quality-of-life outcomes. Beyond skull base extension, SRS may also be considered for patients with small, radiographically stable tumors, significant surgical risk, or a strong preference for noninvasive therapy. However, unlike surgery, SRS does not provide tissue diagnosis. For operable vagal schwannomas, surgery remains the preferred option due to the feasibility of nerve-sparing techniques, whereas SRS may be considered as an alternative for carefully selected patients with higher surgical risk or skull base involvement (5, 15).

As treatment choices in this cohort were guided by tumor operability, anatomical constraints, comorbidities, and patient preference rather than randomization, direct outcome comparisons across treatment groups were not intended. Instead, this study presents a real-world clinical decision-making framework and highlights the complementary roles of surgery and SRS in managing post-styloid PPS tumors.

This study has several limitations. First, its retrospective, single-center design and small sample size limit the generalizability of the findings. Although the rarity of post-styloid PPS neurogenic tumors makes large single-institution series difficult, only 9 patients had intraoperative confirmation of nerve origin. Therefore, the reported diagnostic accuracy and diagnostic performance metrics should be interpreted as descriptive institutional experience rather than definitive validation of the imaging criteria. Second, histological confirmation was limited to surgically treated cases and the small subset of patients who underwent preoperative FNA. In many cases, FNA was deferred because of the deep anatomical location, risk of neurovascular injury, or low diagnostic yield. Patients managed with SRS or observation lacked histopathologic confirmation, which may have introduced diagnostic uncertainty despite typical imaging features and multidisciplinary assessment. Third, follow-up duration was heterogeneous, with a median follow-up of only 12 months. Although no recurrence was observed, this duration may be insufficient to detect late recurrence or delayed functional deterioration, particularly for slow-growing neurogenic tumors. Finally, the 25-year study period may have introduced variability in imaging resolution, neuromonitoring availability, and surgical techniques, and selection bias remains possible because the cohort was drawn from a tertiary referral center.

Given the rarity of post-styloid PPS tumors—where most reported series include only a small number of patients—this study contributes valuable institutional experience with one of the larger single-center cohorts in Taiwan. Our findings emphasize the importance of vessel displacement patterns in predicting nerve origin and guiding nerve-sparing surgical strategies. Future multicenter, prospective studies incorporating standardized imaging protocols and long-term functional outcomes will be essential to further validate and expand upon these observations.

In conclusion, this study suggests that preoperative vessel displacement patterns on routine CT/MRI may help anticipate the nerve of origin and guide surgical strategy for post-styloid PPS tumors. Although imaging is not infallible, its predictive utility may support individualized risk counseling and nerve-sparing approaches whenever feasible. SRS may be considered as an alternative for selected patients with skull base extension or higher surgical risk. Given the small number of surgically confirmed cases and heterogeneous follow-up duration, future multicenter studies with standardized imaging protocols, interobserver validation, and long-term functional outcomes are needed.

## Data Availability

The datasets presented in this article are not readily available because of patient privacy and IRB restrictions. Requests to access the datasets should be directed to the corresponding authors and are subject to institutional approval.

## References

[1] Ijichi K Murakami S. Surgical treatment of parapharyngeal space tumors: a report of 29 cases. Oncol Lett. (2017) 14(3):3249–54. 10.3892/ol.2017.6480 28927073 PMC5588032

[2] Shirakura S Tsunoda A Akita K Sumi T Suzuki M Sugimoto T. Parapharyngeal space tumors: anatomical and image analysis findings. Auris Nasus Larynx. (2010) 37(5):621–5. 10.1016/j.anl.2010.01.003 20185257

[3] Bhardwaj A PS S Sood R Malhotra M Priya M Saikia R. Giant pediatric *de novo* parapharyngeal space pleomorphic adenoma: excision by semi trans-parotid cervical approach. Egypt J Otolaryngol. (2023) 39:57. 10.1186/s43163-023-00428-w

[4] Varoquaux A Fakhry N Gabriel S Garcia S Ferretti A Chondrogiannis S. Retrostyloid parapharyngeal space tumors: a clinician and imaging perspective. Eur J Radiol. (2013) 82(5):773–82. 10.1016/j.ejrad.2013.01.005 23399040

[5] Loperfido A Celebrini A Fionda B Bellocchi G Cristalli G. Diagnostic and therapeutic strategy for vagal schwannoma: case series and literature review. Medicina (Kaunas). (2023) 59(6):1013. 10.3390/medicina59061013 37374217 PMC10303656

[6] Sandler ML Sims JR Sinclair C Sharif KF Ho R Yue LE. Vagal schwannomas of the head and neck: a comprehensive review and a novel approach to preserving vocal cord innervation and function. Head Neck. (2019) 41(7):2450–66. 10.1002/hed.25758 30957342

[7] Furukawa M Furukawa MK Katoh K Tsukuda M. Differentiation between schwannoma of the vagus nerve and schwannoma of the cervical sympathetic chain by imaging diagnosis. Laryngoscope. (1996) 106(12 Pt 1):1548–52. 10.1097/00005537-199612000-00021 8948621

[8] Saito DM Glastonbury CM El-Sayed IH Eisele DW. Parapharyngeal space schwannomas: preoperative imaging determination of the nerve of origin. Arch Otolaryngol Head Neck Surg. (2007) 133(7):662–7. 10.1001/archotol.133.7.662 17638778

[9] Dankle SK. Neoplasms of the parapharyngeal space. Ear Nose Throat J. (1987) 66:491–501. 3428198

[10] Anil G Tan TY. Imaging characteristics of schwannoma of the cervical sympathetic chain: a review of 12 cases. AJNR Am J Neuroradiol. (2010) 31(8):1408–12. 10.3174/ajnr.A2212 20616174 PMC7966108

[11] Graffeo CS Van Abel KM Morris JM Carlson ML Van Gompel JJ Moore EJ. Preoperative diagnosis of vagal and sympathetic cervical schwannomas based on radiographic findings. J Neurosurg. (2017) 126(3):690–7. 10.3171/2016.1.JNS151763 27104848

[12] Thelen J Bhatt AA. Multimodality imaging of paragangliomas of the head and neck. Insights Imaging. (2019) 10(1):29. 10.1186/s13244-019-0701-2 30830483 PMC6399371

[13] Ijichi K Kawakita D Maseki S Beppu S Takano G Murakami S. Functional nerve preservation in extracranial head and neck schwannoma surgery. JAMA Otolaryngol Head Neck Surg. (2016) 142(5):479–83. 10.1001/jamaoto.2016.0113 27032018

[14] Kano H Meola A Yang HC Guo WY Martínez-Alvarez R Martínez-Moreno N. Stereotactic radiosurgery for jugular foramen schwannomas: an international multicenter study. J Neurosurg. (2018) 129(4):928–36. 10.3171/2017.5.JNS162894 29125412

[15] Mendenhall WM Strojan P Beitler JJ Langendijk JA Suarez C Lee AW. Radiotherapy for parapharyngeal space tumors. Am J Otolaryngol. (2019) 40(2):289–91. 10.1016/j.amjoto.2018.12.010 30621929

